# Synergistic Effects of Ambient Temperature and Air Pollution on Health in Europe: Results from the PHASE Project

**DOI:** 10.3390/ijerph15091856

**Published:** 2018-08-28

**Authors:** Antonis Analitis, Francesca de’ Donato, Matteo Scortichini, Timo Lanki, Xavier Basagana, Ferran Ballester, Christopher Astrom, Anna Paldy, Mathilde Pascal, Antonio Gasparrini, Paola Michelozzi, Klea Katsouyanni

**Affiliations:** 1 Department of Hygiene, Epidemiology, and Medical Statistics, Medical School, National and Kapodistrian University of Athens, 11527 Athens, Greece; aanalit@med.uoa.gr; 2 Department of Epidemiology, Lazio Regional Health Service, 00147 Rome, Italy; f.dedonato@deplazio.it (F.d.D.); m.scortichini@deplazio.it (M.S.); p.michelozzi@deplazio.it (P.M.); 3 Department of Health Security, National Institute for Health and Welfare (THL), 70210 Kuopio, Finland; timo.lanki@thl.fi; 4 Institute of Public Health and Clinical Nutrition, University of Eastern Finland, 70210 Kuopio, Finland; 5 Barcelona Institute for Global Health (ISGlobal), 08003 Barcelona, Spain; xavier.basagana@isglobal.org; 6 Department of Experimental and Health Sciences, Universitat Pompeu Fabra (UPF), 08002 Barcelona, Spain; 7 Spanish Consortium for Research on Epidemiology and Public Health (CIBERESP), 28029 Madrid, Spain; ballester_fer@gva.es; 8 FISABIO, Epidemiology and Environmental Health Joint Research Unit, Universitat Jaume I, Universitat de València, 46010 Valencia, Spain; 9 Unit of Occupational and Environmental Medicine, Department of Public Health and Clinical Medicine, Umeå University, 90187 Umeå, Sweden; christofer.astrom@umu.se; 10 National Public Health Institute, Directorate of Public Health, 1097 Budapest, Hungary; paldy.anna@oki.antsz.hu; 11 Department of Environmental Health (DSE), Santé Publique France, 94415 Saint Maurice, France; m.pascal@invs.sante.fr; 12 Department of Public Health, Environments and Society, London School of Hygiene & Tropical Medicine, London WC1H 9SH, UK; Antonio.Gasparrini@lshtm.ac.uk; 13 Centre for Statistical Methodology, London School of Hygiene & Tropical Medicine, London WC1E 7HT, UK; 14 Department of Population Health Sciences and Department of Analytical, Environmental and Forensic Sciences, School of Population Health & Environmental Sciences, King’s College London, London SE1 9NH, UK

**Keywords:** temperature, air pollution, climate change and extreme weather events, interaction, short-term health effect, vulnerability

## Abstract

We studied the potential synergy between air pollution and meteorology and their impact on mortality in nine European cities with data from 2004 to 2010. We used daily series of Apparent Temperature (AT), measurements of particulate matter (PM_10_), ozone (O_3_), and nitrogen dioxide (NO_2_) and total non-accidental, cardiovascular, and respiratory deaths. We applied Poisson regression for city-specific analysis and random effects meta-analysis to combine city-specific results, separately for the warm and cold seasons. In the warm season, the percentage increase in all deaths from natural causes per °C increase in AT tended to be greater during high ozone days, although this was only significant for all ages when all causes were considered. On low ozone days, the increase in the total daily number of deaths was 1.84% (95% CI 0.87, 2.82), whilst it was 2.20% (95% CI 1.28, 3.13) in the high ozone days per 1 °C increase in AT. Interaction with PM_10_ was significant for cardiovascular (CVD) causes of death for all ages (2.24% on low PM_10_ days (95% CI 1.01, 3.47) whilst it is 2.63% (95% CI 1.57, 3.71) on high PM_10_ days) and for ages 75+. In days with heat waves, no consistent pattern of interaction was observed. For the cold period, no evidence for synergy was found. In conclusion, some evidence of interactive effects between hot temperature and the levels of ozone and PM_10_ was found, but no consistent synergy could be identified during the cold season.

## 1. Introduction

There has been an increasing awareness of the acute health effects of temperature extremes, particularly heat, supported by a growing body of scientific evidence [1,2,3,4,5,6]. Recent evidence on the effects of hot weather, such as the high mortality experienced during the extreme heat wave that struck Europe in the summer of 2003, has raised public concern [7,8].

Air pollution is also a well-known public health risk factor. In the past 25 years, results from many epidemiologic studies have given evidence for a positive association between air pollutants concentrations and total and cause-specific mortality [9,10,11]. Large multicity studies in Europe, USA, and other part of the world have documented and quantified the adverse effects of air pollution on health [12,13,14]. Fine particles (PM_10_, PM_2.5_), ozone, nitrogen dioxide, and sulfur dioxide—even at relatively low concentrations—have been linked with increases in morbidity and mortality.

Air pollution concentrations are determined to an extent by prevailing meteorological conditions. For example, as O_3_ is formed by a photochemical reaction, the intensity of solar radiation during heat waves leads to a rise in O_3_ concentrations, whilst the decrease in rainfall may also enhance ambient pollutant concentrations. Climate change could affect air quality directly but also indirectly via changes in human behavior [15]. There is a lot of knowledge available on how current weather does affect air pollutant concentrations. Climate change can affect local to regional air quality directly through changes in chemical reaction rates, boundary layer heights that affect vertical mixing of pollutants, and changes in synoptic airflow patterns that govern pollutant transport. Energy demand could also increase, and as we continue to rely on the combustion of fossil fuels, this could lead to an increase in particulate matter (PM) and NOx levels. Additionally, some particles are products of photochemical reactions and will be higher at higher temperatures at any given precursor emission level.

Subgroups of the population that are most vulnerable to the effects of air pollution or heat are relatively well established [16,17]. They include the elderly; those in institutions, such as residential care homes, who are at particular risk; young children and asthma sufferers; people suffering from chronic diseases, particularly cardiovascular and respiratory conditions, renal diseases, diabetes, and obesity as well as those taking certain medications; people of lower socioeconomic status; and those living in densely populated urban neighborhoods [10].

To better assess the potential impact of current climate change scenarios on human health, it is necessary to understand not only the independent effects of temperature and other meteorological variables (adjusting for confounders, including pollutants) but also to elucidate any synergistic effects between meteorology and air pollution. It is reasonable to hypothesize that any such effects will become more important under extreme conditions that could occur in the future due to greater climate instability.

We report here the study of the potential synergy between air pollution and meteorology, including extreme weather events and their impact on mortality outcomes. These were undertaken within the framework of the PHASE Project (Public Health Adaptation Strategies to Extreme weather events), a European collaboration funded by the European Agency for Health and Consumers (EAHC) within the EU Commission Health Program 2008–2013.

## 2. Data and Methods

In the PHASE project, a large data base including daily values of exposure and health outcome variables was compiled from nine cities spanning across Europe—Athens, Barcelona, Budapest, Helsinki, London, Paris, Rome, Stockholm, and Valencia—within the years 2004–2010. The data included mortality by cause and age group and meteorological and air pollution daily series.

The mortality data consisted of the daily number of deaths from all natural (ICD-9: 1–799; ICD-10: group A–R), cardiovascular (ICD-9: 390–459; ICD-10: group I), and respiratory causes (ICD-9: 460–519; ICD-10: group J) by specific age groups (15–64, 65–74, and >75 years and all ages). Mortality data were obtained from the official registries of each participating city.

The air pollution data included daily concentrations of gaseous and particulate pollutants, specifically, particulate matter with aerodynamic diameter <10 μm (PM_10_, 24-h mean), ozone (O_3_, maximum 8-h moving average) and nitrogen dioxide (NO_2_, mean 24-h). The air pollution series were obtained from the urban monitoring network of each city.

The meteorological data consisted of daily series of air temperature (1-h, °C), dew point temperature (1-h, °C), relative humidity (%), wind speed (m/s) and direction (degrees), and sea level pressure (hPa). The main meteorological exposure variable used was apparent temperature (AT), which is a combination of hourly air temperature (temp) and dew point temperature (dew) using the following formula:

AT = −2.653 + 0.994 × temp + 0.0153 × (dew)^2^

More details about the data and the calculation of AT may be found in De Donato et al. [18] and Kalkstein and Valimont [19].

### Statistical Analysis

The analysis was carried out separately for the warm (April to September) and cold (October to March) periods from the years 2004 to 2010 as well as for each outcome and age group. Based on previous results [3,20], the temperature and AT–mortality association was considered J-shaped in the warm season and linear in the cold season. Furthermore, a priori lags for the effects of AT were chosen. As it is known that heat effects are more immediate, lags 0–3 days were considered for the warm season and for effects of heat waves. Cold-related effects on mortality are more prolonged and have been estimated to last about two weeks [3]. However, as the focus of the present paper is on temperature and pollution interaction, lags 0–6 days were considered for cold period because the effects of pollution are mostly observed at smaller lags. AT max was introduced as exposure variable for the warm period and AT min for the cold period.

The synergy between heat waves and air pollutants was studied for the summer months (June to August). We used the definition of a heat wave developed within the EUROHEAT project [4]. Briefly, heat waves were defined as (1) periods of at least two days with AT exceeding the 90th percentile of the monthly distribution of the particular city or (2) periods of at least two days in which the minimum temperature exceeded the 90th percentile of its respective distribution and AT exceeded the median monthly value. This definition for heat waves, which is described in detail in D’ Ippoliti et al. [4], is based on extensive multi-city analysis and takes into account duration of high temperatures, geographical location, the time of the year, and the importance of both minimum and maximum temperatures.

A two stage statistical analysis plan was followed. For the first stage, i.e., the city-specific analysis, a Generalized Estimating Equations (GEE) modeling approach was applied [21]. We assumed a Poisson distribution for the outcome variable (daily number of deaths). Furthermore, we assumed that the observations within each season of a specific year are correlated while observations in different years are independent. Based on previous studies [2,3], a first order correlation structure was specified.

The equations of the model for (a) warm season, (b) cold season, and (c) heat waves are the following:

(a) logE[Y] = AT_+_ + Pol + AT_+_ × Pol + AT_−_ + [covariates]
(b) logE[Y] = AT + Pol + AT × Pol + [covariates]
(c) logE[Y] = HW + Pol + HW × Pol + [covariates]

where Y is the number of deaths on each day with expectation E[Y].

In the models on synergies of AT and heat waves, AT was entered in the model as a piecewise-linear term with different slopes above (AT_+_) and under (AT_−_) the turning point in the warm period analysis and as a linear term (AT) in the cold one. A dummy variable indicating a heat wave (HW) day was the main exposure variable when heat episodes were studied. Potential confounders introduced as covariates were air pollutants (μg/m^3^, lag 0–1), barometric pressure (linear term, hPa), wind speed (linear term, m/s), calendar month (five dummy variables), day of the week (six dummy variables), holiday (yes/no), and time trend (linear and quadratic term). The air pollutants were considered alternatively in each model. Separate analyses were done for each age group. For the investigation of interaction between temperature/heat wave and pollutant effects, an interaction term between temperature/heat wave and each pollutant separately was introduced in the model. To illustrate the results, the effect of a degree Celsius change in AT was estimated on a “high” and a “low” pollution day. As effect on a “low” pollution day, we presented the combined effects from each city on days with concentration of the pollutant equal to the 25th percentile of the city-specific pollutant distribution; as effect on a “high” pollutant day, we presented the corresponding effect calculated on days with concentrations equal to the 75th percentile of the same distribution. Similarly, the effect of heat wave days was estimated on a “high” and a “low” pollution day, as defined above. At the second stage of analysis, a quantitative summary of all individual-city results (interaction coefficient and effects of AT and heat waves on “low” and “high” pollution) was obtained using random effects meta-analysis according to the DerSimonian and Laird method [22].

As sensitivity analysis, we used the daily temperature and relative humidity measurements instead of AT. We applied the same models in terms of other covariates and lags but instead of AT, we used maximum daily temperature for the warm season and minimum daily temperature for the cold season and relative humidity in all models.

## 3. Results

In Table 1 and Table 2, descriptive characteristics of the outcome and exposure variables in the participating cities are shown. The average total daily number of deaths ranged from 17 in Valencia to 133 in London, mainly reflecting the difference in the city populations. Air pollution and temperature indicators showed a large variability in the air quality and meteorological conditions among cities.

In Table 3, the synergistic effects between maximum AT and O_3_, PM_10_, and NO_2_ are shown for the warm season and for the three outcome variables i.e., all, cardiovascular (CVD), and respiratory number of deaths by age group. In general, the percentage increase in all deaths from natural causes per °C increase in AT tended to be greater during high ozone days, although this was only significant for all ages when all causes were considered. Specifically, on low ozone, days the increase in the total daily number of deaths was 1.84% (95% CI 0.87, 2.82), whilst it is 2.20% (95% CI 1.28, 3.13) in the high ozone days per 1 °C increase in AT. Interaction with PM_10_ was significant for CVD causes of death for all ages and for ages 75+. The increase in the CVD number of deaths for all ages associated with an increase of 1 °C in max AT during days with low PM_10_ was 2.24% (95% CI 1.01, 3.47), whilst it was 2.63% (95% CI 1.57, 3.71) during days with high PM_10_ levels. Significant interaction of AT and PM_10_ levels was also found when persons aged 75+ were considered. No evidence for interaction between AT and NO_2_ was found.

In Table 4, the synergistic effects between heat wave days and O_3_, PM_10_, and NO_2_ are shown. No consistent pattern was observed, and no consistent evidence for interaction was found.

In Table 5, the synergistic effects between minimum AT and O_3_, PM_10_, and NO_2_ in the cold season are shown. No evidence for synergy was found for any of the pollutants and health endpoints analyzed, with the exception of NO_2_ and all natural causes mortality for all ages, where there was a statistically significant interaction and the percentage increase in the daily number of deaths associated with a decrease in minimum apparent temperature by 1 °C was 0.76 (95% CI 0.60, 0.93) on a day with low NO_2_ and 0.88% (95% CI 0.68, 1.08) on a day with high NO_2_.

We also analyzed the data from Mediterranean and North Central cities separately, and the results were consistent with the above patterns (Supplementary Material Figures S1 and S2).

From the sensitivity analysis results, using temperature and relative humidity separately in the models, we found that there were generally larger effects of temperature per °C compared to AT effects. The interaction of temperature with ozone levels for all causes of death for all ages was statistically significant with the increase in the daily number of deaths associated with 1 °C increase in max daily temperature equal to 2.64% (95% CI 0.89, 4.43) in low ozone days and 2.86% (1.16, 4.59) in high ozone days. The interactions observed between temperature and PM_10_ for all ages and among those >75 years for CVD deaths were on the same direction but did not reach the nominal level of statistical significance. Similarly, in the cold season, the interaction between temperature and NO_2_ was not statistically significant but followed the same pattern (Supplementary Material Table S1).

## 4. Discussion

The PHASE project was one of the first multi-city projects to examine potential synergistic effects on mortality between meteorology and air pollution. In the warm season, we found significant interaction between AT and temperature and ozone concentrations for their effects on the total daily number of deaths for all ages. The AT effects appeared to be larger for CVD causes on days with increased PM_10_ concentrations, although not to a statistically significant level in sensitivity analysis. No consistent evidence was found for interaction with air pollution when heat waves were considered. In the cold season, there was no consistent evidence for AT or temperature/air pollution interaction.

There have been few studies addressing the issue of synergy between meteorology and pollution and their effects on health. Among the reported studies, more have investigated hot weather rather than cold weather. Some studies have reported effects of temperature modified by pollutants and some have reported effects of pollutants modified by temperature. However, although the interaction was the same in statistical terms, the results reported did not always allow comparisons by magnitude of effects. The results found in the present analysis of effect modification by PM_10_ and a suggestion for ozone with respect to warm/hot temperatures are consistent with a number of reports in the literature. Thus, Sartor et al. [23] reported synergy with ozone; Parodi et al. [24] and Ren et al. [25] also reported synergy between temperature and ozone exposure for effects on cardiovascular mortality. In a study in Germany, Breitner et al. [26] reported effect modification in the temperature–mortality association by O_3_. Jhun et al. [27] examined the effect modification by temperature on the O_3_ effect; although they did not find a statistically significant interaction, they observed a pattern compatible with some effect modification. Several studies have reported synergy of temperature and PM for effects on total non-accidental deaths and on cardiovascular mortality [28,29,30,31]. In Hefei, China, a study found synergy between PM_10_ concentrations and temperature in their effects on mortality, which was more pronounced in females and in the illiterate [32]. However there have also been studies that have observed no synergy either with PM or O_3_ or both [1,26,33,34]. Part of the inconsistency in the reported results may be attributed to specific characteristics of the cities or areas where the studies were done. For example, population exposure to outdoor temperature and pollution may differ according to air condition use, design aspects of homes, local habits related to opening windows, etc. Another problem can be the fact that analysis methods followed were not consistent and did not allow comparisons. For example, some studies have mutually adjusted for air pollutants, while others used single pollutant models. However, there is accumulating evidence that the warm/hot increasing temperature effects are enhanced by high pollution levels (especially PM_10_ and ozone) and vice versa and that effects of pollutants are enhanced by the presence of high temperature.

Most of the studies conducted so far have examined effects on mortality. However, Lepeule et al. [35] examined the effects of temperature and its interaction with black carbon concentrations on lung function in an elderly cohort of men and found some indication of synergy. This may indicate that the synergistic effects between meteorological variables—most importantly temperature—and health effects, including outcomes other than mortality, deserve more attention. More research on this topic is needed to enhance our understanding of the ways in which these interconnected environmental exposures interact.

As described above, most studies have investigated temperature–mortality interaction and only a few studies have addressed heat waves directly. The lack of identifiable and consistent synergistic effects with heat waves and ozone/PM_10_ in the present study is in contrast with previously reported results from the EuroHEAT project [36]. The analysis of Analitis et al. largely concerned the same cities but for an earlier period (1990 to 2004), which included the major heat wave that occurred in 2003. Another older study in Athens that reported synergistic effects [37] included a major heat wave that occurred in 1987. A reason for this discrepancy may be the fact that no such major heat wave was present after 2004. Additionally, the warmest cities in Europe have developed and implemented prevention plans and thus reduced the effects of heat waves [18]. Although the study of interaction with air pollution concentrations and the occurrence of a heat wave is very important, there is a lack of related results. The investigation is further hindered by the lack of consistent definition of what constitutes a heat wave as well as the limitation in statistical power because heat wave days are relatively rare.

Some studies have looked at the effects of temperature, including the cold season and low temperatures. Burkart et al. [38] produced bivariate response surfaces, including high and low temperatures, in a study in Berlin and Lisbon. They found evidence of interaction of air pollutants concentrations with high temperature on mortality effects but little evidence of interaction during the cold season. Cheng and Kan [39] reported a significant interaction of PM_10_ and O_3_ and cold temperature on mortality outcomes in Shanghai, China. A recent multi-city study in Europe researched eight cities of which five—Athens, Barcelona, Helsinki, Rome, and Stockholm—were overlapping with the present work. However, the study used different city-specific time periods and different analysis methods and emphasized ultrafine particles. The study found interactive effects between pollutants and hot temperatures but only reported an indication of an interaction between low temperature and ultrafines for the cold season [40].

Our study had the advantage of including a large database from diverse locations in Europe representing large climatic variability as well as variability in the air pollution levels and mix. However, our study also had several limitations. Firstly, our study shared the measurement error problems of all ecological studies that use a series of measurements done at fixed sites to represent the daily exposure of the whole population living in the same city. However, the consistency of findings based on the same methods worldwide indicates that there are effects on mortality from outdoor level temperature and air pollution, which may in fact be underestimated because of measurement error [6]. Thus, the investigation of synergy is valid and useful, although the results may be underestimated. Secondly, we did not have data on variables that may act as effect modifiers either characterizing housing conditions, such as the use of air conditioning, or changes in lifestyle as people become aware of the potential consequences of extreme weather conditions.

## 5. Conclusions

In an analysis of data from 2004 to 2010 in nine European cities, we found some evidence of interactive effects between hot temperature and the levels of ozone and PM_10_ on daily deaths but no evidence of such synergy specifically during heat wave days. No consistent synergy could be identified during the cold season.

## Figures and Tables

**Table 1:** Daily number of deaths by cause and age group in the participating cities.

City	Daily Number of Deaths from All Natural Causes for All Ages and by Age Group (Mean, sd)				Daily Number of Deaths from Cardiovascular Causes for All Ages and for Those >75 Years Old (Mean, sd)		Daily Number of Deaths from Respiratory Causes for All Ages and for Those >75 Years Old (Mean, sd)	
	All Ages	15–64 Years	65–74 Years	>75 Years	All Ages	>75 Years	All Ages	>75 Years
Athens	79.6 (12.4)	12.1 (3.7)	13.7 (4.1)	53.4 (9.9)	36.7 (7.9)	27.1 (6.5)	8.4 (3.4)	6.6 (3.0)
Barcelona	37.8 (8.5)	4.9 (2.3)	5.6 (2.5)	27.3 (7.0)	12.0 (4.2)	9.8 (3.7)	4.3 (2.7)	4.2 (2.9)
Budapest	64.2 (10.1)	14.5 (4.0)	13.2 (3.7)	34.4 (7.0)	30.9 (6.9)	21.5 (5.5)	3.0 (1.9)	1.6 (1.3)
Helsinki	17.8 (4.5)	4.0 (2.0)	3.1 (1.8)	10.6 (3.5)	7.1 (2.7)	4.9 (2.2)	1.0 (1.0)	0.6 (0.8)
London	132.9 (19.5)	23.8 (5.2)	22.7 (5.4)	85.5 (14.9)	47.0 (9.7)	33.1 (7.5)	19.5 (7.0)	15.1 (5.8)
Paris	103.4 (14.1)	23.3 (4.9)	15.2 (4.1)	63.5 (11.0)	26.3 (5.9)	20.2 (5.2)	6.5 (3.1)	5.1 (2.7)
Rome	58.0 (10.0)	7.5 (2.8)	10.3 (3.4)	39.9 (8.1)	23.5 (5.9)	18.9 (5.2)	3.6 (2.1)	3.0 (1.9)
Stockholm	25.4 (5.4)	3.2 (1.8)	3.4 (1.9)	18.6 (4.5)	10.4 (3.4)	8.7 (3.0)	1.8 (1.4)	1.4 (1.2)
Valencia	16.8 (4.8)	2.7 (1.7)	2.8 (1.7)	11.2 (3.8)	5.5 (2.6)	4.2 (2.2)	2.0 (1.6)	1.7 (1.4)

**Table 2:** Descriptive characteristics of maximum apparent temperature (AT) and pollutant variables in each participating city.

City	AT (Max, °C) Mean (IQR *)	O_3_ (μgm^−3^) Mean (IQR)	PM_10_ (μgm^−3^) Mean (IQR)	NO_2_ (μgm^−3^) Mean (IQR)
Warm Season				
Athens	30.0 (24.6, 35.4)	87.5 (75.1, 100.9)	38.6 (27.7, 44.0)	62.5 (48.7, 73.7)
Barcelona	25.5 (20.8, 30.7)	55.2 (43.0, 65.0)	37.5 (26.0, 45.8)	32.8 (22.0, 42.0)
Budapest	22.3 (17.7, 27.0)	-	30.7 (21.6, 37.9)	34.7 (25.4, 42.7)
Helsinki	15.3 (10.6, 20.1)	73.5 (60.3, 84.2)	16.3 (10.0, 19.5)	23.7 (16.6, 29.3)
London	18.4 (15.0, 21.6)	48.6 (35.4, 60.3)	21.4 (14.8, 26.4)	36.4 (27.2, 44.1)
Paris	20.5 (16.6, 24.2)	80.2 (63.3, 93.4)	23.0 (16.0, 27.5)	29.9 (21.2, 36.8)
Rome	26.4 (20.9, 31.5)	98.3 (84.2, 111.3)	32.0 (25.2, 37.3)	52.0 (41.2, 61.9)
Stockholm	15.9 (11.6, 20.2)	75.2 (62.9, 85.6)	16.2 (10.2, 19.0)	30.1 (23.1, 36.7)
Valencia	29.4 (24.9, 34.5)	61.6 (55.1, 67.2)	55.0 (43.9, 64.1) **	49.0 (38.0, 58.5)
Cold Season				
Athens	9.3 (5.5, 12.7)	46.5 (32.8, 58.6)	36.6 (23.1, 44.2)	60.0 (48.4, 70.4)
Barcelona	6.6 (2.5, 9.3)	24.8 (13.0, 34.4)	39.3 (26.0, 49.0)	47.1 (35.0, 57.0)
Budapest	−0.8 (−4.4, 2.5)	-	41.8 (25.9, 54.6)	42.7 (32.0, 51.0)
Helsinki	−5.1 (−9.4, −1.7)	51.2 (40.6, 61.9)	14.4 (8.8, 17.4)	27.5 (18.5, 33.5)
London	2.4 (−1.2, 5.6)	24.0 (8.5, 36.8)	23.7 (16.5, 28.1)	48.2 (36.6, 57.6)
Paris	2.9 (−0.7, 6.0)	37.7 (21.4, 51.8)	25.2 (16.0, 30.4)	44.1 (34.1, 52.7)
Rome	4.8 (0.5, 8.3)	43.2 (22.8, 60.4)	39.1 (26.0, 49.3)	66.7 (56.9, 76.2)
Stockholm	−3.3 (−6.8, −0.1)	53.9 (43.7, 64.4)	13.6 (8.2, 16.1)	31.5 (23.2, 38.6)
Valencia	6.1 (1.9, 9.8)	36.5 (27.0, 45.2)	55.8 (40.3, 65.5) **	63.5 (51.2, 74.7)

* IQR: interquartile range of the city specific distribution (25th, 75th percentile); ** based on measurements for total suspended particles (TSP).

**Table 3:** Pooled (from nine city-specific estimates) percentage increase (95% CI) in the daily number of deaths per degree Celsius increase in apparent temperature in the warm period in days with “low” (at the 25th percentile of each city-specific distribution) or “high” (at the corresponding 75th percentile) level of pollutant. Results from random effects meta-analysis.

	**% Increase (95% CI) in the Daily Number of Deaths from All Natural Causes Associated with 1 °C Increase in Apparent Temperature on Days with:**					
**Age Group**	**Low O_3_**	**High O_3_**	**Low PM_10_**	**High PM_10_**	**Low NO_2_**	**High NO_2_**
15–64 years	0.13 (−0.37, 0.64)	0.33 (−0.30, 0.97)	0.34 (−0.60, 1.29)	0.51 (−0.34, 1.36)	0.53 (−0.21, 1.29)	0.89 (0.00, 1.78)
65–74 years	1.12 (0.13, 2.11)	1.41 (0.48, 2.36)	2.02 (0.88, 3.18)	1.73 (1.02, 2.45)	2.20 (1.09, 3.33)	1.55 (0.93, 2.16)
75+ years	2.18 (1.04, 3.34)	2.65 (1.56, 3.76)	2.54 (1.48, 3.62)	2.77 (1.87, 3.67)	3.01 (1.83, 4.20)	2.58 (1.70, 3.47)
All ages	**1.84 (0.87, 2.82)**	**2.20 (1.28, 3.13)**	2.26 (1.33, 3.21)	2.30 (1.56, 3.05)	2.51 (1.51, 3.53)	2.16 (1.43, 2.90)
	**% Increase (95% CI) in the Daily Number of Deaths from Cardiovascular Causes Associated with 1 °C Increase in Apparent Temperature on Days with:**					
**Age Group**	**Low O_3_**	**High O_3_**	**Low PM_10_**	**High PM_10_**	**Low NO_2_**	**High NO_2_**
15–64 years	0.48 (−0.60, 1.57)	0.90 (−0.43, 2.26)	0.70 (−0.43, 1.83)	1.34 (0.28, 2.42)	0.73 (−0.32, 1.80)	1.83 (0.52, 3.14)
65–74 years	**1.54 (0.26, 2.84)**	**1.32 (−0.24, 2.90)**	2.46 (0.64, 4.32)	1.88 (0.43, 3.35)	2.66 (0.95, 4.39)	1.73 (0.12, 3.37)
75+ years	2.35 (1.00, 3.71)	2.66 (1.45, 3.88)	**2.26 (0.99, 3.53)**	**2.85 (1.73, 3.98)**	3.11 (1.72, 4.53)	2.68 (1.59, 3.79)
All ages	2.23 (1.00, 3.47)	2.40 (1.23, 3.59)	**2.24 (1.01, 3.47)**	**2.63 (1.57, 3.71)**	2.86 (1.53, 4.20)	2.49 (1.44, 3.54)
	**% Increase (95% CI) in the Daily Number of Deaths from Respiratory Causes Associated with 1 °C Increase in Apparent Temperature on Days with:**					
**Age Group**	**Low O_3_**	**High O_3_**	**Low PM_10_**	**High PM_10_**	**Low NO_2_**	**High NO_2_**
15–64 years	1.94 (−1.38, 5.38)	1.63 (−0.35, 3.65)	1.39 (−1.36, 4.22)	1.24 (−0.71, 3.23)	2.38 (0.35, 4.45)	1.68 (−0.08, 3.47)
65–74 years	3.29 (−1.38, 8.19)	1.96 (−1.13, 5.14)	2.67 (−0.13, 5.56)	3.19 (0.38, 6.07)	3.29 (0.36, 6.31)	3.78 (0.66, 7.00)
75+ years	2.55 (0.95, 4.18)	3.36 (1.68, 5.07)	3.90 (2.02, 5.82)	3.59 (2.20, 4.99)	3.98 (2.14, 5.85)	3.23 (1.81, 4.67)
All ages	2.84 (0.98, 4.74)	3.40 (1.70, 5.13)	4.32 (2.19, 6.49)	3.76 (2.20, 5.34)	4.13 (2.06, 6.24)	3.43 (1.98, 4.91)

Note: in bold where the interaction term is statistically significant at the 0.05 level.

**Table 4:** Pooled (from nine city-specific estimates) percentage increase (95% CI) in the daily number of deaths in days with a heat wave and with “low” (at the 25th percentile of each city-specific distribution) or “high” (at the corresponding 75th percentile) level of pollutant. Results from random effects meta-analysis.

	**% Increase (95% CI) in the Daily Number of Deaths from All Natural Causes on Days with Heat-Waves and:**					
**Age Group**	**Low O_3_**	**High O_3_**	**Low PM_10_**	**High PM_10_**	**Low NO_2_**	**High NO_2_**
15–64 years	7.06 (0.66, 13.87)	4.96 (−0.70, 10.94)	2.86 (−3.52, 9.67)	4.34 (−1.19, 10.18)	4.67 (−0.51, 10.12)	7.81 (2.24, 13.68)
65–74 years	3.23 (−2.78, 9.61)	5.29 (1.08, 9.68)	4.69 (−1.41, 11.16)	6.86 (2.80, 11.08)	6.21 (1.08, 11.60)	8.33 (2.52, 14.46)
75+ years	10.74 (3.70, 18.26)	10.66 (7.01, 14.43)	10.25 (5.94, 14.72)	11.19 (7.62, 14.88)	14.03 (9.69, 18.53)	13.41 (8.52, 18.52)
All ages	8.96 (2.90, 15.37)	8.72 (6.02, 11.48)	7.97 (5.05, 10.97)	9.18 (6.10, 12.36)	10.40 (6.51, 14.43)	11.37 (7.07, 15.85)
	**% Increase (95% CI) in the Daily Number of Deaths from Cardiovascular Causes on Days with Heat-Waves and:**					
**Age Group**	**Low O_3_**	**High O_3_**	**Low PM_10_**	**High PM_10_**	**Low NO_2_**	**High NO_2_**
15–64 years	7.51 (−4.75, 21.35)	6.96 (−4.39, 19.66)	−5.13 (−21.00, 13.92)	1.60 (−9.85, 14.51)	**−2.61 (−14.83, 11.36)**	**8.73 (−2.05, 20.70)**
65–74 years	10.58 (−0.58, 22.99)	5.09 (−2.20, 12.91)	7.74 (−4.84, 21.99)	6.54 (−2.84, 16.82)	10.05 (−0.83, 22.12)	6.69 (−4.66, 19.40)
75+ years	13.60 (3.92, 24.18)	11.24 (5.60, 17.18)	9.55 (2.65, 16.91)	10.93 (5.45, 16.70)	13.95 (6.60, 21.82)	13.37 (6.45, 20.74)
All ages	12.33 (3.67, 21.71)	9.18 (4.31, 14.27)	7.51 (1.76, 13.59)	8.69 (3.28, 14.38)	10.80 (4.34, 17.66)	11.55 (4.78, 18.75)
	**% Increase (95% CI) in the Daily Number of Deaths from Respiratory Causes on Days with Heat-Waves and:**					
**Age Group**	**Low O_3_**	**High O_3_**	**Low PM_10_**	**High PM_10_**	**Low NO_2_**	**High NO_2_**
15–64 years	51.58 (−13.06, 164.28)	4.44 (−17.11, 31.59)	1.80 (−29.18, 46.33)	−0.76 (−19.04, 21.64)	8.68 (−15.17, 39.23)	7.39 (−9.62, 27.60)
65–74 years	2.30 (−17.32, 26.56)	15.29 (−6.60, 42.31)	11.66 (−10.87, 39.90)	23.77 (2.05, 50.11)	22.30 (0.07, 49.48)	24.61 (3.61, 49.86)
75+ years	15.93 (6.23, 26.51)	17.90 (11.12, 25.09)	16.10 (5.77, 27.44)	15.22 (6.95, 24.13)	19.40 (10.43, 29.09)	18.61 (9.43, 28.55)
All ages	13.58 (4.97, 22.90)	16.99 (10.82, 23.51)	13.90 (4.40, 24.26)	18.18 (9.16, 27.94)	21.10 (12.02, 30.93)	20.43 (12.63, 28.76)

Note: in bold where the interaction term is statistically significant at the 0.05 level.

**Table 5:** Pooled (from nine city-specific estimates) percentage increase (95% CI) in the daily number of deaths per degree Celsius decrease in apparent temperature in the cold period in days with “low” (at the 25th percentile of each city-specific distribution) or “high” (at the corresponding 75th percentile) level of pollutant. Results from random effects meta-analysis.

	**% Increase (95% CI) in the Daily Number of Deaths from All Natural Causes Associated with 1 °C Decrease in Apparent Temperature on Days with:**					
**Age Group**	**Low O_3_**	**High O_3_**	**Low PM_10_**	**High PM_10_**	**Low NO_2_**	**High NO_2_**
15–64 years	0.60 (0.21, 0.99)	0.46 (0.16, 0.75)	0.40 (0.16, 0.65)	0.52 (0.30, 0.75)	0.46 (0.17, 0.75)	0.64 (0.35, 0.93)
65–74 years	0.67 (0.25, 1.09)	0.73 (0.43, 1.02)	0.68 (0.29, 1.08)	0.80 (0.47, 1.14)	0.73 (0.45, 1.01)	0.88 (0.46, 1.31)
75+ years	0.90 (0.66, 1.14)	0.82 (0.54, 1.09)	0.92 (0.68, 1.16)	0.81 (0.59, 1.04)	0.85 (0.62, 1.08)	0.95 (0.70, 1.20)
All ages	0.79 (0.60, 0.99)	0.75 (0.55, 0.95)	0.80 (0.60, 1.00)	0.76 (0.58, 0.94)	**0.76 (0.60, 0.93)**	**0.88 (0.68, 1.08)**
	**% Increase (95% CI) in the Daily Number of Deaths from Cardiovascular Causes Associated with 1 °C Decrease in Apparent Temperature on Days with:**					
**Age Group**	**Low O_3_**	**High O_3_**	**Low PM_10_**	**High PM_10_**	**Low NO_2_**	**High NO_2_**
15–64 years	0.95 (0.12, 1.79)	1.14 (0.17, 2.12)	0.65 (−0.02, 1.32)	0.76 (0.21, 1.31)	0.68 (−0.05, 1.41)	0.99 (0.22, 1.78)
65–74 years	1.32 (0.75, 1.90)	1.52 (1.01, 2.04)	1.17 (0.72, 1.62)	1.43 (0.96, 1.90)	1.32 (0.88, 1.76)	1.40 (0.97, 1.82)
75+ years	1.14 (0.87, 1.41)	1.07 (0.83, 1.31)	1.11 (0.75, 1.47)	0.98 (0.70, 1.27)	1.03 (0.74, 1.33)	1.14 (0.82, 1.45)
All ages	1.13 (0.84, 1.43)	1.09 (0.85, 1.34)	1.10 (0.74, 1.47)	1.04 (0.76, 1.32)	1.05 (0.78, 1.32)	1.17 (0.84, 1.50)
	**% Increase (95% CI) in the Daily Number of Deaths from Respiratory Causes Associated with 1 °C Decrease in Apparent Temperature on Days with:**					
**Age Group**	**Low O_3_**	**High O_3_**	**Low PM_10_**	**High PM_10_**	**Low NO_2_**	**High NO_2_**
15–64 years	0.67 (−1.00, 2.38)	0.41 (−1.55, 2.42)	0.99 (−0.73, 2.74)	0.83 (−0.47, 2.16)	0.66 (−0.70, 2.04)	1.10 (−0.50, 2.72)
65–74 years	0.78 (−0.25, 1.82)	0.96 (−0.05, 1.98)	1.01 (0.07, 1.96)	1.12 (0.27, 1.97)	1.15 (0.23, 2.09)	1.40 (0.47, 2.33)
75+ years	0.90 (0.31, 1.49)	0.99 (0.47, 1.51)	1.10 (0.42, 1.79)	0.99 (0.51, 1.48)	**0.79 (0.27, 1.31)**	**1.18 (0.66, 1.71)**
All ages	0.68 (0.05, 1.31)	0.78 (0.08, 1.48)	1.00 (0.37, 1.63)	0.91 (0.43, 1.39)	**0.80 (0.34, 1.26)**	**1.10 (0.53, 1.67)**

Note: in bold where the interaction term is statistically significant at the 0.05 level.

## Supplementary Material

The following are available online at http://www.mdpi.com/1660-4601/15/9/1856/s1, Figure S1: Pooled percent increase (95% Confidence Intervals-CI) in the daily number of deaths by cause and age group, per degree Celsius increase in max apparent temperature in the warm period, in days with “low” (at the 25th percentile of each city-specific distribution) or “high” (at the corresponding 75th percentile) level of pollutant in the Mediterranean (Athens, Barcelona, Rome, Valencia) and North-central (Budapest, Helsinki, London, Paris, Stockholm) cities. Results from random effects meta-analysis, Figure S2: Pooled percent increase (95% Confidence Intervals-CI) in the daily number of deaths by cause and age group, per degree Celsius decrease in min apparent temperature in the cold period, in days with “low” (at the 25th percentile of each city-specific distribution) or “high” (at the corresponding 75th percentile) level of pollutant in the Mediterranean (Athens, Barcelona, Rome, Valencia) and North-central (Budapest, Helsinki, London, Paris, Stockholm) cities. Results from random effects meta-analysis, Table S1: Sensitivity analysis: Pooled (from 9 city-specific estimates) percent increase (95% Confidence Intervals-CI) in the daily number of deaths per degree Celsius change in temperature in days with “low” (at the 25th percentile of each city-specific distribution) or “high” (at the corresponding 75th percentile) level of pollutant. Results from random effects meta-analysis (in bold where the interaction term is statistically significant at the 0.05 level).
