# Supplementary material for: Synergistic Effects of Ambient Temperature and Air Pollution on Health in Europe: Results from the PHASE Project
Source: Int J Environ Res Public Health. 2018 Aug 28;15(9):1856. doi: 10.3390/ijerph15091856 (PMC6163671; doi:10.3390/ijerph15091856)
Supplement: Supplementary file 1 [file ijerph-15-01856-s001.pdf]

Article

Synergistic effects of ambient temperature and air pollution on health in Europe: results from the PHASE project

Supplementary Material

Warm period
Mediterranean cities

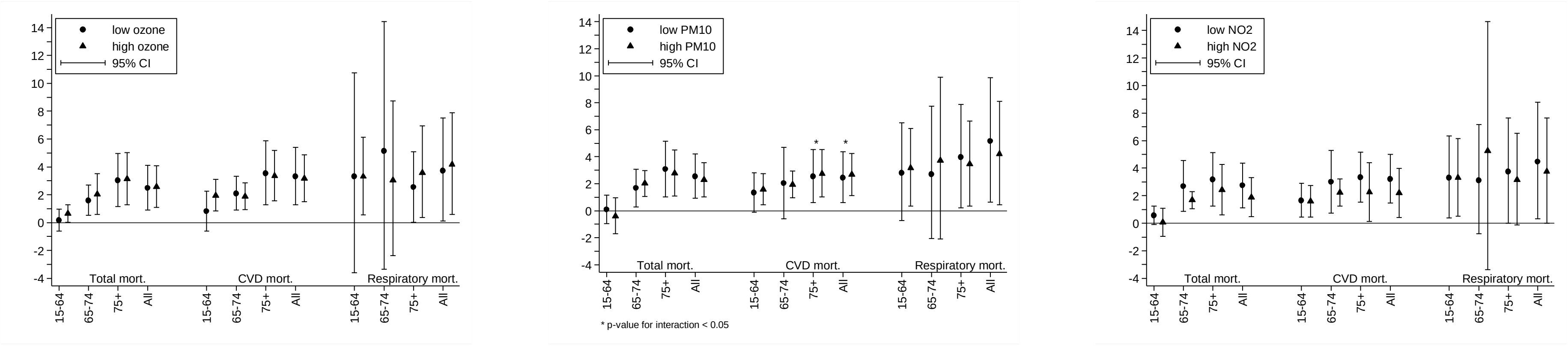

North-central cities

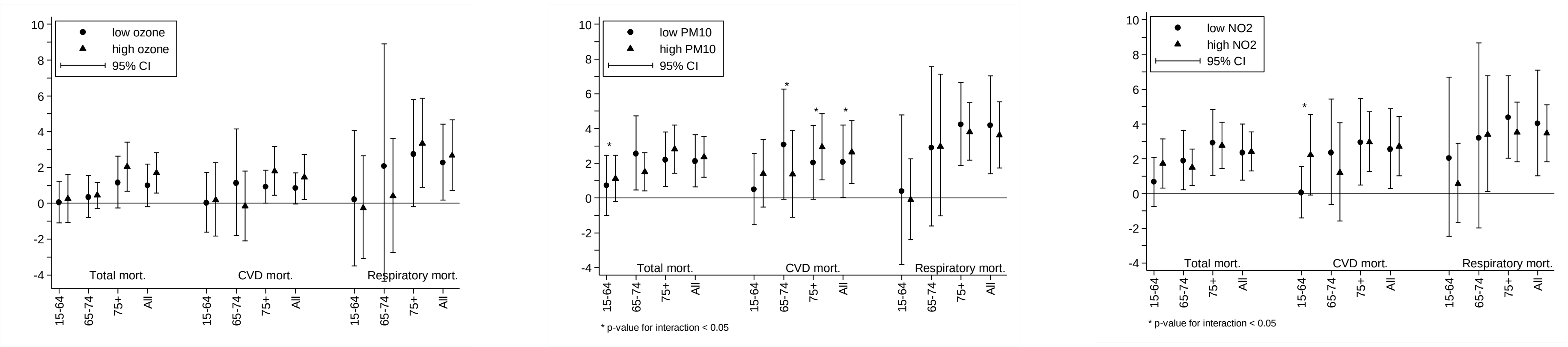

Figure S1. Pooled percent increase (95% Confidence Intervals-CI) in the daily number of deaths by cause and age group, per degree Celsius increase in max apparent temperature in the warm period, in days with "low" (at the 25th percentile of each city-specific distribution) or "high" (at the corresponding 75th percentile) level of pollutant in the Mediterranean (Athens, Barcelona, Rome, Valencia) and North-central (Budapest, Helsinki, London, Paris, Stockholm) cities. Results from random effects meta-analysis.

Cold period
Mediterranean cities

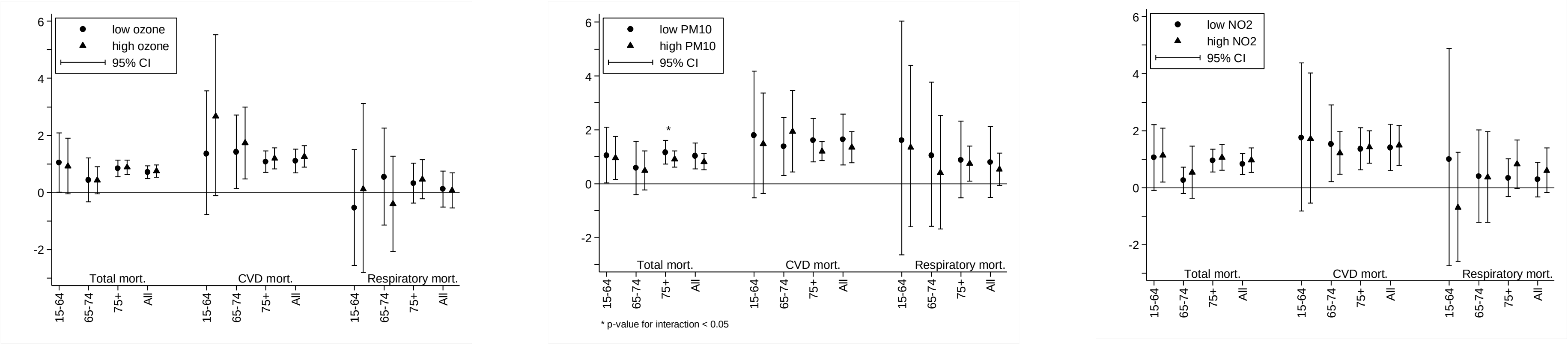

North-central cities

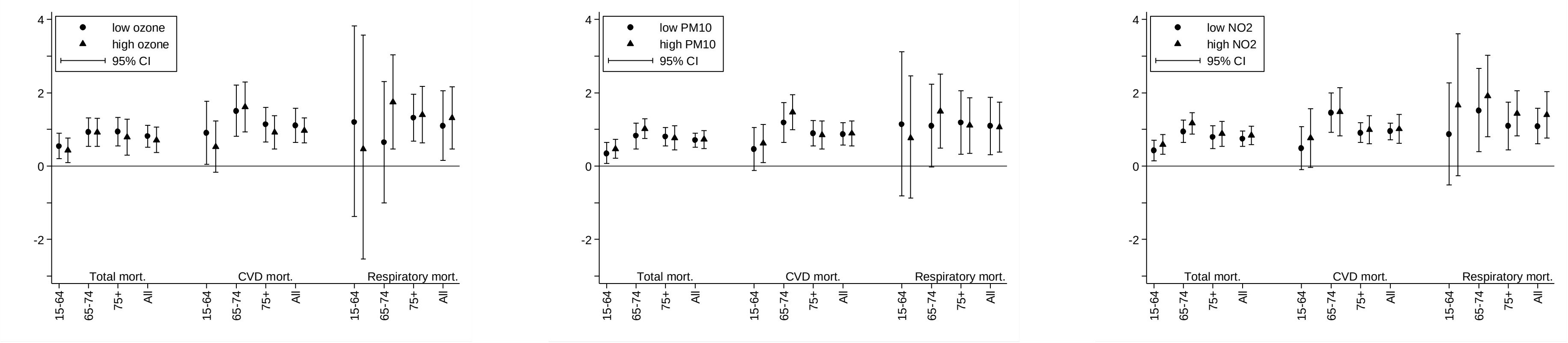

Figure S2. Pooled percent increase (95% Confidence Intervals-CI) in the daily number of deaths by cause and age group, per degree Celsius decrease in min apparent temperature in the cold period, in days with "low" (at the 25th percentile of each city-specific distribution) or "high" (at the corresponding 75th percentile) level of pollutant in the Mediterranean (Athens, Barcelona, Rome, Valencia) and North-central (Budapest, Helsinki, London, Paris, Stockholm) cities. Results from random effects meta-analysis.

Table S1. Sensitivity analysis: Pooled (from 9 city-specific estimates) percent increase (95% Confidence Intervals-CI) in the daily number of deaths per degree Celsius change in temperature in days with "low" (at the 25th percentile of each city-specific distribution) or "high" (at the corresponding 75th percentile) level of pollutant. Results from random effects meta-analysis (in bold where the interaction term is statistically significant at the 0.05 level).

**Warm Period**	**% increase (95% CI) in the daily number of deaths from all natural causes associated with 1°C increase in max temperature on days with:**					
Age group	Low O_3	High O_3	Low PM_{10}	High PM_{10}	Low NO_2	High NO_2
75+ yrs	3.13 (1.01, 5.30)	3.40 (1.40, 5.45)	3.51 (1.63, 5.42)	3.53 (2.01, 5.07)	4.04 (1.99, 6.13)	3.25 (1.73, 4.79)
All ages	**2.64 (0.89, 4.43)**	**2.86 (1.16, 4.59)**	3.03 (1.43, 4.66)	2.92 (1.66, 4.20)	3.35 (1.62, 5.10)	2.73 (1.46, 4.02)
	% increase (95% CI) in the daily number of deaths from cardiovascular causes associated with 1°C increase in max temperature on days with:					
Age group	Low O_3	High O_3	Low PM_{10}	High PM_{10}	Low NO_2	High NO_2
75+ yrs	3.61 (0.85, 6.45)	3.41 (0.94, 5.95)	3.34 (1.07, 5.67)	3.64 (1.68, 5.63)	4.24 (1.79, 6.76)	3.39 (1.41, 5.42)
All ages	3.37 (0.84, 5.97)	3.21 (0.80, 5.67)	3.07 (0.91, 5.27)	3.34 (1.46, 5.26)	3.78 (1.47, 6.14)	3.19 (1.28, 5.14)
**Cold period**	% increase (95% CI) in the daily number of deaths from all natural causes associated with 1°C decrease in min temperature on days with:					
Age group	Low O_3	High O_3	Low PM_{10}	High PM_{10}	Low NO_2	High NO_2
75+ yrs	1.08 (0.82, 1.35)	1.04 (0.63, 1.45)	1.08 (0.72, 1.45)	0.97 (0.64, 1.30)	1.04 (0.72, 1.36)	1.11 (0.78, 1.44)
All ages	0.92 (0.68, 1.17)	0.93 (0.58, 1.28)	0.94 (0.63, 1.25)	0.90 (0.62, 1.18)	0.92 (0.68, 1.16)	1.01 (0.73, 1.28)
	% increase (95% CI) in the daily number of deaths from cardiovascular causes associated with 1°C decrease in min temperature on days with:					
Age group	Low O_3	High O_3	Low PM_{10}	High PM_{10}	Low NO_2	High NO_2
75+ yrs	1.27 (0.95, 1.59)	1.28 (0.82, 1.74)	1.25 (0.77, 1.74)	1.12 (0.71, 1.53)	1.22 (0.84, 1.60)	1.30 (0.88, 1.73)
All ages	1.26 (0.91, 1.62)	1.34 (0.88, 1.80)	1.22 (0.74, 1.70)	1.16 (0.75, 1.57)	1.24 (0.89, 1.59)	1.36 (0.93, 1.80)
